# Social participation of community-dwelling older adults in western China: A latent profile analysis

**DOI:** 10.3389/fpubh.2022.874204

**Published:** 2022-08-22

**Authors:** Di Luo, Shiqi Yu, Jun Wang, Ying Zhu, Lining Yang, Ruonan Bai, Qianyi Rao, Qiang Zhang, Di Wu, Feng Wang, Qinghua Zhao, Mingzhao Xiao

**Affiliations:** ^1^Department of Urology, The First Affiliated Hospital of Chongqing Medical University, Chongqing, China; ^2^Department of Rehabilitation Medicine, The First Affiliated Hospital of Chongqing Medical University, Chongqing, China; ^3^Service Center of Rehabilitation Assistive Technology of Sichuan Province, Sichuan, China; ^4^Inner Mongolia Autonomous Region Rehabilitation Assistive Device Center, Inner Mongolia, China; ^5^Panzhihua Wuyue Technology Co., Ltd, Pan Zhihua, China; ^6^Department of Nursing, The First Affiliated Hospital of Chongqing Medical University, Chongqing, China

**Keywords:** social participation, aged, latent profiles analysis, western China, activities of daily living

## Abstract

**Objective:**

Social participation has become a policy framework to address population aging. However, little is known about the social participation of older adults in western China, and extensive, multicenter, regional research is lacking. This research investigated the profiles of social participation of older adults in western China and explored the characteristics and factors influencing social participation.

**Method:**

This cross-sectional study was conducted in 3 provinces (Chongqing, Sichuan, and Inner Mongolia) in western China from March 2021 to December 2021 and included 3,456 participants aged 60 years or older. Social participation was assessed using the Chinese version of the Impact on Participation and Autonomy Questionnaire (IPA). Latent profile analysis (LPA) was performed to extract latent classes of social participation among older adults in western China. The chi-square test and multinomial regression analyses were used to identify differences between these classes.

**Results:**

Three social participation classes were identified by LPA: high social participation (25.2%), moderate social participation (55.1%), and low social participation (19.7%). Being older, having a primary school education level, having mobility or speaking impairment, using assistive devices, and having a chronic disease were highly associated with the low social participation class (*P* < 0.05). Furthermore, older adults with no dependence (OR = 0.018, 95% CI = 0.005–0.062) or mild dependence (OR = 0.039, 95% CI = 0.011–0.139) in activities of daily living (ADLs) were less likely to be in the low social participation class. Older adults who were cared for by non-spouse primary caregivers were more likely to be assigned to the moderate social participation group (OR = 2.097, 95% CI = 1.501–2.930) than to the high social participation group.

**Conclusions:**

Most older adults in western China have a moderate level of social participation. Advanced age, reduced ADL ability, reduced speech ability, reduced mobility, and non-spouse care are related to the level of social participation. Healthcare professionals should pay attention to the predictors for different classes, identifying high-risk groups as early as possible.

## Introduction

China, an “aging giant”, has approximately 264 millio people over 60 years old (18.7%) and 191 million people over 65 years old (13.5%) (1). How to cope well with aging has become a key challenge with the intensification of population aging. Social participation has been regarded as a vital factor in the active response to population aging for a long time (2, 3). Social participation is a broad concept and can take many forms, including informal social participation, e.g., having contact with friends, and formal social participation, e.g., attending religious organizations, and other forms of social participation such as volunteering, paying money for services or caring for another person (4). In 1987, social participation was deemed as an important component of successful aging (5). In 2002, the World Health Organization (WHO) proposed that “health, participation, and security” were the three pillars of active aging (6). In 2015, the concept of active aging was replaced by healthy aging as the basis for the lead policy framework (7), and social participation was still emphasized within environmental needs and as one of the strategies is “to remove barriers to participation” (8). Overall, the social participation of older adults is a key factor in successfully addressing with aging worldwide.

Regarding social participation, previous studies have focused on the antecedents, experiences, or outcomes of social participation and emphasized the importance of examining who participates as well as where and when they engage (9). Ensuring cognitive function (10), ensuring better mental health (11), preventing physical prefrailty (12) and reducing long-term care costs (13) have been revealed to be associated with social participation among older adults. Moreover, the impacts of psychology [having positive attitudes about aging (14), having a high sense of community (15)], and physical disabilities [surviving a stroke (16) and being frail (17)] on social participation have been explored. Previous studies have also found individual differences in different types of social participation and various influencing factors (12, 18). However, previous studies have widely discussed the influencing factors of social participation, but traditionally, these studies have focused on the analysis of the relationship between variables while ignoring the people-centered discussion of individual differences in social participation.

Latent profile analysis (LPA) is a person-centered algorithm that models heterogeneity by classifying individuals into unobserved groupings (latent classes) with similar (more homogenous) patterns (19). LPA enables us to explain internal relationships with indiscrete manifest variables and categorize individuals into common profiles. In recent years, LPA has been widely used to analyze profile constructs (20–22). Thus, LPA may be a significant complement to traditional variable-entered approaches and construct an optimized multidimensional perspective of social participation.

According to the International Classification of Functioning, Disability, and Health (ICF) framework, the environment is the key factor affecting social participation (23). Although national surveys based on large databases in China (24, 25), and a survey of representative cities in some regions about social participation have been carried out, little is known about social participation among older adults in western China. Moreover, compared to eastern China, western China has a relatively underdeveloped economy and a more serious aging situation, with a larger population (27.12%), a higher old-age dependency ratio (15.34%), and lower healthy aging (1, 26, 27).

In sum, to better respond to population aging challenges and identify and develop targeted interventions, this study intends to (a) explore social participation subgroups among community-dwelling older adults in western China by LPA and (b) identify the influencing factors, differences, and similarities among subgroups.

## Methods

### Participants

This cross-sectional study was conducted in 3 provinces in western China from March 2021 to December 2021 and included 3,456 participants aged 60 years or older. In the first stage, considering the purpose of the study, the representative provinces, and the feasibility of the research area, we selected three provinces (Chongqing, Sichuan, and Inner Mongolia) and five cities (Chongqing, Panzhihua, Nanchong, Deyang, and Hohhot) in western China. In the second stage, one community was randomly selected from each city as the research site. At the final stage, based on the number of older adults registered in the local community office, we conducted a cluster sample and expected to include 5,943 older adults.

Eligible people who met the inclusion criteria were randomly selected. The inclusion criteria were (1) age ≥60 years old; (2) registered permanent residence in the districts selected; and (3) usual residence in the districts selected. The exclusion criteria were (1) a history of dementia, mental deficiency, or other psychiatric diseases; (2) residence in long-term care facilities; and (3) older adults or primary caregivers refusing to be investigated. Finally, 2,314 older adults refused to participate, and 3,629 subjects were recruited, for a response rate of 61.06%. After the exclusion of responses with incomplete answers to the questionnaire, a total of 3,456 subjects were included in the final analysis.

### Procedures

In this study, face-to-face interviews were carried out by trained investigators. Each participant completed three parts of the questionnaire, including personal information, assessment of social participation, and assessment of the activities of daily living (ADLs). Personal information and social participation were self-assessed. If participants were unable to read, the investigators read the questions aloud to them, and the questionnaire was completed according to the statements of the subjects with their permission. Assessment of the ADLs was conducted by trained investigators through observation and inquiry based on uniform questionnaire items.

The survey was conducted as follows: (a) a survey was conducted in the vicinity of the street office with the consent of the community street management department; (b) a local residential area was randomly selected for a door-to-door investigation; and (c) the places where older adults usually visited were chosen.

Before the formal investigation, the participants were informed about the purpose and content of the survey by trained investigators. If the participants chose to complete the survey, consent was presumed. If participants experienced physical fatigue or discomfort during the interview, the interview was suspended or terminated. The research was approved by the Ethics Committee of the First Affiliated Hospital of Chongqing Medical University (NO. 2020-622).

### Measures

#### Personal information

The section on personal information included two parts. The first part collected sociodemographic data, e.g., gender, age, education level, monthly income level, marital status, number of offspring, and primary caregiver. The second part collected health data, including the cumulative number of chronic diseases (hypertension, diabetes, heart disease, or other chronic diseases reported by participants), self-reported physical function impairment (vision, hearing, speech, mobility), and use of assistive devices (yes or no).

#### The activities of daily living

The ability to function independently in ADLs was measured by the Barthel Index (28). The following 10 items were rated: eating, bathing, dressing, grooming, controlling bowel function, controlling bladder function, using the toilet unaided, transferring, walking, and stair climbing. A lower score indicates a lower ability to carry out the ADL. According to the total score, ADL ability was divided into four levels: no dependence (100 points), mild dependence (61–99 points), moderate dependence (41–60 points), and severe dependence ( ≤ 40 points) (29).

#### Social participation

The Impact on Participation and Autonomy Questionnaire (IPA), which is a self-report instrument that measures people's perceptions of participation and autonomy and can be used in a variety of populations, was used in this study. The original English scale was developed by Cardol et al. (30), while Li et al. (31) created the Chinese version and revised it in 2013. The scale contains 25 items and has 4 dimensions: autonomy indoors (7 items), family role (7 items), autonomy outdoors (5 items), and social life (6 items). Autonomy indoors mainly refers to bodily activity in the indoor environment and the ability for self-care with independence or assistance. The family role dimension is related to the responsibilities and obligations of the family. Autonomy outdoors mainly involves activity in the outdoor environment, the possibility of controlling leisure time, and the opportunity to meet with relatives and friends. The dimension of social life mainly refers to autonomy in social life and social relations and the possibility of helping others. A 5-point Likert scale is adopted, where “a lot” is assigned 0 points and “a little” is assigned 4 points. A higher score indicates a lower level of social participation. In the present study, the Cronbach's α for this scale was 0.976.

### Statistical analysis

LPA was carried out using the IPA's 25 items as indicators and was conducted using the Mplus version 8.3. This study used the following fit indices to select the optimal number of profiles: the Bayesian information criterion (BIC), the Akaike information criterion (AIC), the value of Sample Size-Adjusted BIC (aBIC) and the entropy test for model evaluation; the Lo–Mendell–Rubin likelihood ratio test (LMR), the bootstrapped likelihood ratio test (BLRT) for model comparison. Lower BIC, AIC, aBIC and higher entropy indicate better fit. A significant *p*-value on LMR means the solution with *k* number of classes is better than the *k*-1 classes. The BLRT mainly compares the fitting differences between *k*-1 and *k* class models. The theoretical base for class solutions was also considered in selecting the best number of participant classes.

IBM SPSS 20.0 was used to analyze personal information and the ADL and IPA data with the chi-square test and multinomial logistic regression. Descriptive analyses were initially conducted to characterize the sample. Descriptive statistics for the demographic variables were calculated, including the median and interquartile range for continuous variables, such as the IPA total and subscale scores. Frequency counts and percentages were used to summarize categorical variables, including gender, marital status, and education level.

## Results

### Characteristics of the participants

Table 1 presents the characteristics of the participants. Of the 3,456 participants, 1,886 (54.6%) were female, 1,570 (45.4%) were male, and those aged 70~79 were predominant (42.2%). Only 20.7% of the participants reported a high school or above education, and more than half of the participants' monthly income was 1,000~2,999 yuan. A total of 82.6% of the participants lived with their spouse, and 57.6% reported their spouse as their primary caretaker. Most participants were not dependent in their ADLs (88.2%); approximately one-fifth of the study participants reported impaired vision (22.7%) or impaired hearing (20.1%), with a small number of participants reporting impaired mobility (12.0%) or impaired speech (8.7%) without using assistive devices (65.5%).

**Table 1:** Descriptive characteristics of the participants (*n* = 3,456).

**Demographic characteristic**	***N***	**Percentage**
Gender		
Male	1,570	45.40%
Female	1,886	54.60%
Age (group)		
60~	1,438	41.60%
70~	1,458	42.20%
80~	560	16.20%
Education level		
Illiteracy	398	11.50%
Elementary school	1,477	42.70%
Elementary school	865	25.00%
High school or above	716	20.70%
Job		
Farmer	1,100	31.80%
Worker	1,725	49.90%
Other	631	18.30%
Marital status		
Living with spouse	2,855	82.60%
Living without spouse	601	17.40%
Monthly income		
<1,000 RMB	893	25.80%
1,000~2,999 RMB	1,797	52.00%
≥3,000 RMB	766	22.20%
Primary caretaker		
Spouse	1,989	57.60%
Non-spouse	1,203	34.80%
Live alone	264	7.60%
Self-report Impaired vision		
Yes	786	22.70%
No	2,670	77.30%
Self-report impaired hearing		
Yes	696	20.10%
No	2,760	79.90%
Self-report impaired mobility		
Yes	414	12.00%
No	3,042	88.00%
Self-report impaired speech		
Yes	299	8.70%
No	3,157	91.30%
Number of somatic chronic conditions		
0	1,376	39.80%
1	1,931	55.90%
≥2	149	4.30%
Use assistive devices		
Yes	1,193	34.50%
No	2,263	65.50%
ADL		
No dependence	3,048	88.20%
Mild dependence	284	8.20%
Moderate and severe dependence	124	3.60%

### Latent profiles analysis of IPA

**Figure 1:**
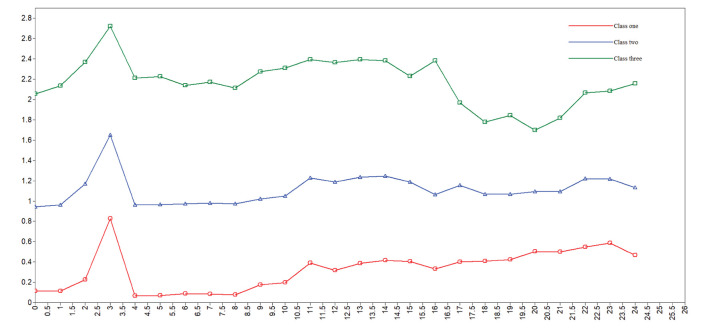
The distribution of three potential classes of social participation.

Table 2 shows fit indices for latent profile models 1–5. The AIC, BIC, and aBIC, which were used to test the goodness of fit, decreased continuously from class 1 to class 5 but declined the fastest at class 3, dropping to the lowest for class 5. The entropy, ranging between 0.981 and 0.985, showed an optimal fit for the five models. The 2-class model was excluded because the high AIC, BIC and aBIC; the 4-class model was excluded because of the lowest entropy; and the 5-class model was excluded because of the non-significant LMR. Furthermore, the distribution of case number and class probability in the 3-class model was much more reasonable. In summary, the 3-class model was determined to be the best-fitting model. Figure 1 shows the distribution of three potential classes with different participation levels.

**Table 2:** Model fit indices for different models.

**Models**	**AIC**	**BIC**	**aBIC**	**LMR**	**BLRT**	**Entropy**	**Category probability**	**Case** **number**
1-class	215504.646	215812.039	215653.165	————	————	————	1	3456
2-class	173801.837	174269.075	174027.587	<0.001	<0.001	0.985	0.730/0.270	2524/932
**3-class**	**153328.679**	**153955.761**	**153631.659**	**<0.001**	**<0.001**	**0.982**	**0.252/0.551/0.197**	**872/1903/681**
4-class	144038.964	144825.891	144419.174	<0.001	<0.001	0.981	0.235/0.208/0.511/0.046	812/718/1768/158
5-class	137292.607	138239.378	137750.047	0.353	<0.001	0.983	0.170/0.124/0.475/0.193/0.038	588/428/1640/667/133

AIC, Akaike Information Criterion; BIC, Bayesian Information Criterion; aBIC, adjusted Bayesian Information Criterion; LMR, Lo-Mendell-Rubin likelihood ratio test; BLRT, Bootstrap likelihood ratio test.

### Characteristics of classes

Table 3 presents the IPA scores reflecting social participation for each group. Table 4 shows the different characteristics of the subjects in each group. According to the results of the LPA analysis, we compared the IPA scores reflecting social participation in three classes. The characteristics of each group were analyzed from the chi-square test results. Except for gender and marital status, each personal information, disease characteristics, and ADL score was significantly different among the three classes (*P* < 0.01).

**Table 3:** Four dimensions for different models.

**Dimensions**	**Class one** **(*n* = 872)** **[M (P25, P75)]**	**Class two** **(*n* = 1,903)** **[M (P25, P75)]**	**Class three** **(*n* = 681)** **[M (P25, P75)]**	**Total sample** **(*n* = 3,456)** **[M (P25, P75)]**
IPA total scores	8 [1, 14]	25 [25, 30]	50 [49, 58]	25 [19, 38]
Autonomy indoors	0 [0, 1]	7 [7, 7]	14 [14, 16]	7 [2, 9]
Family role	1 [0, 4]	7 [7, 9]	14 [14, 18]	7 [6, 12]
Autonomy outdoors	2 [0, 4]	5 [5, 7]	11 [10, 13]	5 [4, 9]
Social relations	1 [0, 6]	6 [6, 7]	12 [10, 12]	6 [6, 9]

Class one: “high social participation”; Class two: “moderate social participation”; Class three: “low social participation”.

**Table 4:** Characteristics of individuals in different potential groups based on participation level by the latent profile analysis (*n* = 3,456).

**Variables**	**Class one** **(*n* = 872)**	**Class two** **(*n* = 1,903)**	**Class three** **(*n* = 681)**	***χ2***	***P-*value**
Gender				1.005	0.605
Male	400 (45.9%)	851 (44.7%)	319 (46.8%)		
Female	472 (54.1%)	1,052 (55.3%)	362 (53.2%)		
Age (group)				134.142	<0.01
60~	**486 (55.7%)**	749 (39.4%)	203 (29.8%)		
70~	314 (36.0%)	822 (43.2%)	322 (47.3%)		
80~	72 (8.3%)	332 (17.4%)	156 (22.9%)		
Education level				32.606	<0.01
Illiteracy	120 (13.8%)	210 (11.0%)	68 (10.0%)		
Elementary school	330 (37.8%)	839 (44.1%)	**308 (45.2%)**		
Middle school	240 (27.5%)	492 (25.9%)	133 (19.5%)		
High school or above	182 (20.9%)	362 (19.0%)	172 (25.3%)		
Job				169.052	<0.01
Farmer	364 (41.7%)	594 (31.2%)	142 (20.9%)		
Worker	401 (46.0%)	863 (45.3%)	**461 (67.7%)**		
Other	107 (12.3%)	446 (23.4%)	78 (11.5%)		
Marital status				3.437	0.179
Living with spouse	717 (82.2%)	1,590 (83.6%)	548 (80.5%)		
Living without spouse	155 (17.8%)	313 (16.4%)	**133 (19.5%)**		
Monthly income				75.159	<0.01
<1,000 RMB	289 (33.1%)	461 (24.2%)	143 (21.0%)		
1,000~2,999 RMB	424 (48.6%)	**1,054 (55.4%)**	319 (46.8%)		
≥3,000 RMB	159 (18.2%)	388 (20.4%)	219 (32.2%)		
Offspring				15.01	<0.01
0	10 (1.1%)	23 (1.2%)	**13 (1.9%)**		
1	294 (33.7%)	578 (30.4%)	255 (37.4%)		
≥2	568 (65.1%)	1,302 (68.4%)	413 (60.6%)		
Primary caretaker				56.488	<0.01
Spouse	**562 (64.4%)**	1,008 (53.0%)	419 (61.5%)		
Non-spouse	228 (26.1%)	761 (40.0%)	214 (31.4%)		
Live alone	82 (9.4%)	134 (7.0%)	48 (7.0%)		
Self-report impaired vision				28.015	<0.01
Yes	173 (19.8%)	407 (21.4%)	**206 (30.2%)**		
No	699 (80.2%)	1,496 (78.6%)	475 (69.8%)		
Self-report impaired hearing				112.174	<0.01
Yes	115 (13.2%)	349 (18.3%)	**232 (34.1%)**		
No	757 (86.8%)	1,554 (81.7%)	449 (65.9%)		
Self-report impaired mobility				136.455	<0.01
Yes	84 (9.6%)	160 (8.4%)	**170 (25.0%)**		
No	788 (90.4%)	1,743 (91.6%)	511 (75.0%)		
Self-report impaired speech				147.339	<0.01
Yes	17 (1.9%)	151 (7.9%)	**131 (19.2%)**		
No	855 (98.1%)	1,752 (92.1%)	550 (80.8%)		
Number of chronic diseases				348.515	<0.01
0	**499 (57.2%)**	777 (40.8%)	100 (14.7%)		
1	311 (35.7%)	1,057 (55.5%)	563 (82.7%)		
≥2	62 (7.1%)	69 (3.6%)	18 (2.6%)		
Use assistive devices				92.476	<0.01
Yes	211 (24.2%)	658 (34.6%)	**324 (47.6%)**		
No	661 (75.8%)	1,245 (65.4%)	357 (52.4%)		
ADL				357.79	<0.01
No dependence	**816 (93.6%)**	1,753 (92.1%)	479 (70.2%)		
Mild dependence	53 (6.1%)	126 (6.6%)	105 (15.4%)		
Moderate and severe dependence	3 (0.3%)	24 (1.3%)	98 (14.4%)		

Class one, “high social participation”, comprised 25.2% (872/3,456) of the sample. This class had low IPA scores, indicating a high level of social participation (M (P25, P75) = 8[1, 14]). Moreover, the older people in this group were relatively younger, had more offspring, were taken care of by their spouses, and had better health status than those in other groups.

Class two, “moderate social participation”, had the highest proportion of the sample, with 55.1% (1,903/3,456). This class showed relatively poorer social participation in this study (M [P25, P75] = 25[25, 30]). The majority of older adults were in this class, and most people had a moderate monthly income (1,000~2,999 yuan).

Class three, “low social participation”, represented 19.7% (681/3,456) of the sample. The IPA score of class three was the highest, twice that of class two and six times that of class one, indicating that class three had the lowest level of social participation among the three classes (M [P25, P75] = 50[49, 58]). Moreover, class three had the most subjects living without a spouse, while older adults having no offspring were the largest in this class. In addition, the proportion of subjects with impaired vision, impaired hearing, impaired mobility, and impaired speech was the largest in class three.

### Multinomial logistics regression

Table 5 presents the multinational logistic regression results. Compared with class one (high social participation), older adults with non-spouse primary caregivers (OR = 2.097, 95% CI: 1.501–2.930) or without impaired mobility (OR = 1.407, 95% CI: 1.014–1.951) were more likely to be assigned to class two (moderate social participation). Meanwhile, older adults with no impaired speech (OR = 0.282, 95% CI: 0.160–0.495) or without assistive devices (OR = 0.605, 95% CI: 0.492–0.745) were less likely to be assigned to class two.

**Table 5:** Multinomial logistic regression on IPA subgroups.

**Variable**	**Class two**		**Class three**	
	**OR**	**95%CI**	**OR**	**95%CI**
Education (elementary school)	1.389*	1.067–1.809	2.163**	1.531–3.056
Aged				
60~	0.381**	0.279–0.521	0.374**	0.252–0.556
70~	0.592**	0.435–0.805	0.662*	0.454–0.965
Month income (1,000~2999 RMB)	1.379**	1.084–1.755	0.757	0.561–1.023
Job				
Famer	0.429**	0.309–0.596	0.36**	0.223–0.583
Worker	0.527**	0.406–0.685	2.177**	1.496–3.167
Impaired mobility (No)	1.407*	1.014–1.951	0.676*	0.463–0.985
Impaired speech (No)	0.282**	0.160–0.495	0.179**	0.098–0.328
Use assistive devices (No)	0.605**	0.492–0.745	0.622**	0.469–0.824
Primary caretaker (non-spouse)	2.097**	1.501–2.930	1.572	0.986–2.504
Number of chronic diseases (1)	2.294**	1.523–3.457	4.711**	2.387–9.295
ADL				
No dependence	0.361	0.103–1.258	0.018**	0.005–0.062
Mild dependence	0.314	0.087–1.136	0.039**	0.011–0.139

*P < 0.05, **P < 0.01; Reference group: Class one; OR: Odds ratio; 95%CI: 95% Confidence Interval.

Older adults with no impaired mobility (OR = 0.676, 95% CI: 0.463–0.985), no impaired speech (OR = 0.179, 95% CI: 0.098–0.328), no dependence (OR = 0.018, 95% CI: 0.005–0.062), and mild dependence (OR = 0.039, 95% CI: 0.011–0.139) and those without assistive devices (OR = 0.622, 95% CI: 0.469–0.824) were less likely to be assigned to class three (low social participation) than class one.

## Discussion

By using LPA, the present study identified three distinct classes of social participation among community-dwelling older adults in western China, that is, high social participation (class one), moderate social participation (class two), and low social participation (class three), with distribution rates of 25.2, 55.1, and 19.7%, respectively. Moreover, it explored the characteristics and influencing factors of these groups, which was expected to assist health practitioners in identifying older adults who are at risk of low social participation and devising intervention strategies.

This study revealed that improving the social participation of community older adults in western China is urgent. The proportion of individuals with high participation (25.2%) was lower than that in Switzerland (29.5%) (22) and near to that in the Netherlands (25.9%) (32) and rural China (26%) (33). The difference may be attributed to different methods of measuring social participation or regional differences. Compared with other more developed areas in China, 41.2% of older adults in Shanghai participated in at least 3 leisure activities, and approximately 36.5% of the 41.2% self-reported successful aging (34). Social participation is not only an independent health behavior closely related to aging but also an indispensable link in the strategy to deal with population aging. Therefore, we should encourage more older adults to engage in social participation.

Furthermore, more than half of the older adults were assigned to the moderate social participation class in this study, and nearly 20% were assigned to the low social participation class. Considering that social participation is increasingly recognized as a modifiable determinant of health and wellbeing (22), it is vital to identify subgroups that should be particularly emphasized to maximize the maintenance of social participation or even improve the level of social participation to prevent further decline in the level of participation. However, the number of institutions and organizations that provide social engagement services to senior citizens declined in China from 2010 to 2017 (35). In 2016, the number of community service agencies in eastern China (237,393) was almost double that in western China (94,563) (1). Platforms are expected to provide conditions for older adults to participate in society, which enables strengthening the participation environment and actively isolating older adults to carry out formal or informal activities.

In this study, mobility and speech were found to be essential physical functions for older adults to actively communicate with the outside world and also extremely important abilities in the process of social participation. Based on the connotation of social participation, the essence of social participation needs communication and interaction, which may be one of the reasons (36). Moreover, this study also found that older adults in the moderate social participation class did not necessarily have mobility impairment, while those in the low social participation class were more likely to have mobility impairment than those in the high participation class. On the one hand, according to the ICF framework, individual, social, and environmental factors all interact to shape social participation in a complex and dynamic way (37). In addition to personal health status, personal interests and personal awareness of social participation will have an impact on social participation (38, 39). On the other hand, there may be an interaction between social participation and personal health status. Low levels of physical activities are a significant risk factor associated with functional decline (40), and decreased social participation may carry the risk of decreased physical function (41). In turn, social participation is also critical to the success of postponing mobility disability (42). For example, low-key social participation has emerged as a vital form of social participation for disabled older adults (43). This may highlight the necessity and particularity of social participation in later life and suggest that social participation is important for people with different dysfunction.

In this study, having a non-spouse caregiver was independently associated with the moderate social participation group compared with the high social participation group. Spouses typically take on the role of care, especially when an older adult has a sickness or functional dependency. Furthermore, older adults with a spouse may have better ADL ability than those without a spouse (44). A retrospective longitudinal study in Japan indicated that individuals who lived alone or with a spouse were less likely to continue to receive home care than those who lived with others (45). In addition, this study showed that, although living with a spouse was associated with better social participation, older adults could experience a decline in social participation when living with their spouse but being taken care of by someone other than their spouse, for example, their offspring or housekeeper, which deserves to be taken into account when carrying out an intervention.

In addition, the present study found that those who did not use assistive devices were less likely to be assigned to the moderate or low social participation classes. Individuals who use assistive devices may have some dysfunction, which may affect their social participation. In fact, rehabilitation assistive devices can compensate for the dysfunction of body parts or replace body parts, which is an effective measure for older adults and disabled groups to engage in social participation. A study in Canada found that people who used vision and mobility aids were less likely to participate in social activities than those who did not use such aids (46). However, the cause and effect in these studies were unclear. Some studies hold that the use of assistive devices has a positive impact on social participation. Rousseau et al. (47) found that following wheelchair acquisition, social participation significantly improved, although confounding variables might also have played a role. Conversely, a systematic review summarized measures to promote social participation and found inadequate supportive materials to enhance social participation (48). Therefore, considering that many factors may affect the extent to which assistive devices affect social participation, such as fit, comfort, type, function, acceptability, and even stigma, it is necessary to further control for confounding factors and clarify the relationship between them. Moreover, even devices with good performance must adapt to the use environment and meet the needs of users (49). In sum, the impact of assistive devices on social participation requires further investigation.

## Limitations

This study has several limitations. First, the current study is a cross-sectional descriptive study, limiting its ability to make a causal argument. A longitudinal study is needed to further investigate the interaction between health factors and social participation. Second, there are 12 provinces in western China, and we only selected 3 provinces and cities with representative characteristics, including Chongqing, with mountainous characteristics; Inner Mongolia, with a vast territory; and Sichuan, with a complex and diverse terrain. More provinces and people in western China will be included to increase the representativeness of the sample in future research. Additionally, considering that agreeing to complete questionnaires is also a form of social participation, those who agreed to complete the questionnaire may have had certain social participation abilities. Although we carried out household surveys to find older adults who could not go out, we may still have missed some subjects with low social participation levels. In the future, more comprehensive results should be obtained through information monitoring or other means of assessment. Finally, we cannot rule out confounders because of certain unmeasured parameters that may influence social participation and level changes and stratification.

## Conclusions

In sum, as an overview of types of social participation through a person-centered approach, this study sheds light on the heterogeneity of social participation and clearly identifies three classes of social participation among older adults. The majority of older adults have a moderate level of social participation. Being over 80 years old, having mobility impairment, having speech impairment, and having a non-spouse primary caregiver may affect the level of social participation of older adults. In the future, to improve the level of social participation, we should focus on old-older adults, protect the ADL ability of older adults, and reduce the mobility and communication barriers of older adults.

## Data availability statement

The original contributions presented in the study are included in the article/supplementary material, further inquiries can be directed to the corresponding author/s.

## Ethics statement

The studies involving human participants were reviewed and approved by the Ethics Committee of the First Affiliated Hospital of Chongqing Medical University (NO.2020-622) and all the participants provided oral consent and willingness to complete the investigation.

## Author contributions

MX and QZhao: conceptualization, supervision, and funding acquisition. DL, RB, QR, QZhan, DW, and FW: investigation. DL and SY: statistical analyses and writing of the paper. DL, SY, JW, YZ, and LY: revision and editing of the paper. All authors contributed to the article and approved the submitted version.

## Funding

This research was supported by the National Key R&D Program of China (2020YFC2005900), and the Science and Technology Committee of Chongqing, China (cstc2020jscx-cylhX0002). The funders had no role in the study design, data collection, analysis, interpretation, manuscript writing, or decision to submit the manuscript.

## Conflict of interest

Author FW is employed by Panzhihua Wuyue Technology Co., Ltd. The remaining authors declare that the research was conducted in the absence of any commercial or financial relationships that could be construed as a potential conflict of interest.

## Publisher's note

All claims expressed in this article are solely those of the authors and do not necessarily represent those of their affiliated organizations, or those of the publisher, the editors and the reviewers. Any product that may be evaluated in this article, or claim that may be made by its manufacturer, is not guaranteed or endorsed by the publisher.
